# A Rare Case of Herpes Esophagitis in an Immunocompetent Elderly Patient

**DOI:** 10.7759/cureus.21854

**Published:** 2022-02-03

**Authors:** Ryuichi Ohta, Chiaki Sano

**Affiliations:** 1 Communiy Care, Unnan City Hospital, Unnan, JPN; 2 Community Medicine Management, Shimane University Faculty of Medicine, Izumo, JPN

**Keywords:** family medicine, general medicine, rural hospital, acyclovir, elderly, immunocompetent, herpes esophagitis

## Abstract

Herpes esophagitis is common among immunocompromised hosts but is relatively rare among immunocompetent patients. Its symptoms are vague because many different symptoms can be induced by esophageal lesions. Here, we report a case of herpes esophagitis in an elderly immunocompetent patient. A 91-year-old woman visited our community hospital with a complaint of appetite loss for several days. Although she did not have any symptoms of epigastric, oral, or retrosternal pain, multiple ulcers were detected in her esophagus. Biopsy of the edge of the ulcer showed giant cells, indicating a herpes virus infection. She was diagnosed with herpes esophagitis and treated with acyclovir for one week. Her symptoms completely resolved after treatment, and she was discharged. Herpes esophagitis can manifest as vague symptoms in immunocompetent elderly patients. Therefore, herpes esophagitis must be considered in the differential diagnosis of elderly patients presenting with vague symptoms.

## Introduction

Herpes esophagitis is relatively common among immunocompromised patients who have undergone organ transplantation and use antitumor drugs [1]. Symptoms include chest pain, dysphasia, and nausea/vomiting. Prompt diagnosis is important for the effective treatment of herpes esophagitis because of weak host immunity. The main treatment includes the administration of antiviral drugs, such as acyclovir. Clinicians should consider this disease when there are symptoms related to the gastrointestinal organs, such as the esophagus and stomach, in immunocompromised hosts.

However, herpes esophagitis is rare in immunocompetent patients [2]. Normally, humans can suppress viral proliferation through innate and acquired immunity. Most viral infections can be suppressed and self-limited in immunocompetent patients. Mental and physical stress can decrease patients’ immunity, which can cause herpes infections of the skin. However, systemic viral infections, such as pneumonia, esophagitis, and hepatitis, are uncommon. There are few reports regarding herpes esophagitis among immunocompetent patients [3,4].

Herpes esophagitis among older people can be atypical and difficult to diagnose because of ambiguous symptoms. We present the case of an elderly woman with appetite loss who was eventually diagnosed with herpes esophagitis. This case suggests that herpes esophagitis is a possible differential diagnosis among elderly patients with vague symptoms.

## Case presentation

A 91-year-old independent woman visited our hospital with the chief complaint of appetite loss for several days. Seven days before admission, she felt fatigued without a fever, impinging on her life. Three days before admission, her appetite decreased. These symptoms gradually worsened, causing more fatigue and drowsiness. Her medical history included hypertension, dyslipidemia, and gastric cancer that had been treated 20 years before. Her medications included 5 mg amlodipine and 2.5 mg atorvastatin.

On the day of admission, her Glasgow Coma Scale score was 14 (E3V5M6), and her vital signs were as follows: blood pressure 154/80 mmHg, pulse rate 86 beats per minute, respiratory rate 21 breaths per minute, body temperature 37.1°C, and oxygen saturation 96% in room air. No other abnormalities were detected on physical examination. The laboratory data are presented in Table 1.

**Table 1:** Initial laboratory data. Ig: immunoglobulin; HSV: herpes simplex virus

Marker	Level	Range
White blood cells	7.2	3.5–9.1 × 10^3^/μL
Neutrophils	87.4	44.0–72.0%
Lymphocytes	6.6	18.0–59.0%
Monocytes	5.9	0.0–12.0%
Eosinophils	0	0.0–10.0%
Basophils	0.1	0.0–3.0%
Red blood cells	3.28	3.76–5.50 × 10^6^/μL
Hemoglobin	9.8	11.3–15.2 g/dL
Hematocrit	42.0	33.4–44.9%
Mean corpuscular volume	84.8	79.0–100.0 fL
Platelets	37.7	13.0–36.9 × 10^4^/μL
Erythrocyte sedimentation rate	32	2–10 mm/hour
Total protein	6.9	6.5–8.3 g/dL
Albumin	3.5	3.8–5.3 g/dL
Blood sugar	108	70-109 mg/dL
Total bilirubin	1.1	0.2–1.2 mg/dL
Aspartate aminotransferase	18	8–38 IU/L
Alanine aminotransferase	11	4–43 IU/L
Alkaline phosphatase	204	106–322 U/L
Lactate dehydrogenase	240	121–245 U/L
Blood urea nitrogen	11.2	8–20 mg/dL
Creatinine	0.61	0.40–1.10 mg/dL
Serum Na	135	135–150 mEq/L
Serum K	2.9	3.5–5.3 mEq/L
Serum Cl	89	98–110 mEq/L
Creatine kinase	45	56–244 U/L
C-reactive protein	7.62	<0.30 mg/dL
Thyroid-stimulating hormone	1.77	0.35–4.94 μIU/mL
Free T4	1.1	0.70–1.48 ng/dL
IgG	1,470	<135 mg/dL
IgA	546	
IgM	127	
HSV IgG	55.4	<2.0 S/CO
HSV IgM	0.17	<0.80 S/CO
HIV antibody	0.00	<0.99 S/CO
Urine test		
Leucocytes	(-)	
Nitrite	(-)	
Protein	(-)	
Glucose	(-)	
Urobilinogen	(-)	
Bilirubin	(-)	
Ketone	(-)	
Blood	(-)	
pH	5.5	
Specific gravity	1.018	
Fecal occult blood	(-)	

**Figure 1:**
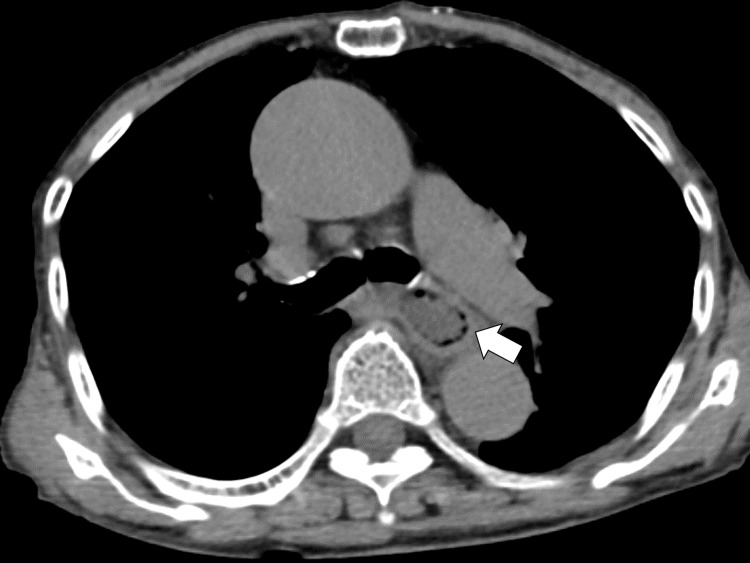
Computed tomography of the chest shows gas-accumulated lesions on the wall of the esophagus.

There were no noteworthy abnormalities in immunoglobulins, thyroid function, or total protein and albumin levels. Magnetic resonance imaging of the brain showed no abnormalities, except for atrophy caused by aging. To rule out systemic cancers, computed tomography was performed, which revealed that there was no typical lymphadenopathy or mass indicating cancer; rather, there were gas-accumulated lesions on the wall of the esophagus (Figure 1).

**Figure 2:**
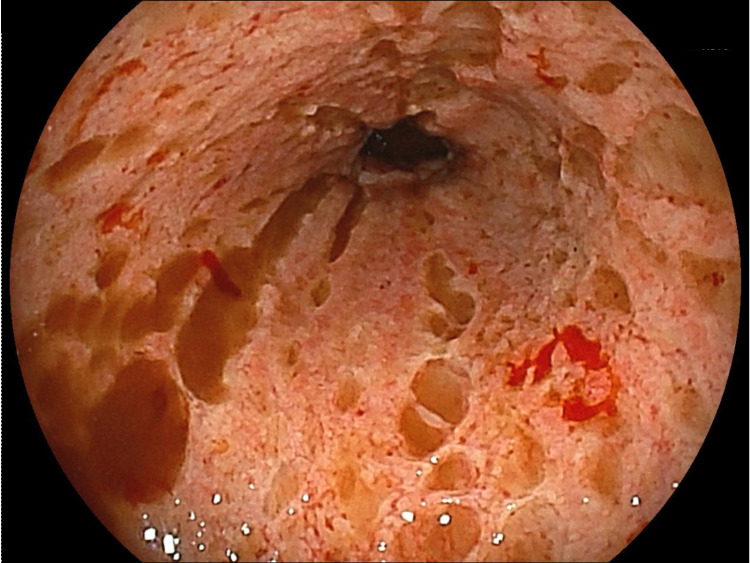
Upper gastrointestinal endoscopy reveals multiple round ulcers and erosions in the esophagus.

On the day after admission, upper gastrointestinal endoscopy revealed multiple round ulcers and erosions in the esophagus (Figure 2).

**Figure 3:**
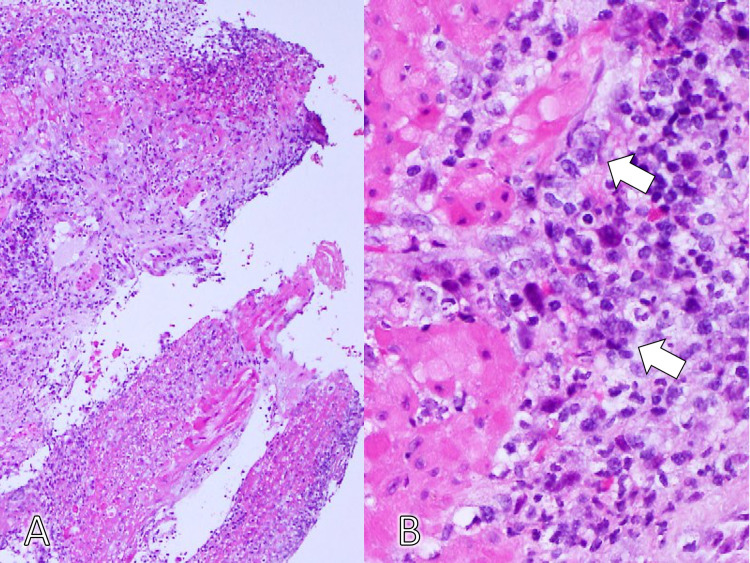
The histopathological finding of the edge of the ulcer shows giant cells with ground-glass nuclei and margination of the chromatin, indicating herpes virus infection (hematoxylin and eosin stain, A: ×40, B: ×400).

Biopsy of the edge of the ulcer showed giant cells, indicating a herpes virus infection (Figure 3).

**Figure 4:**
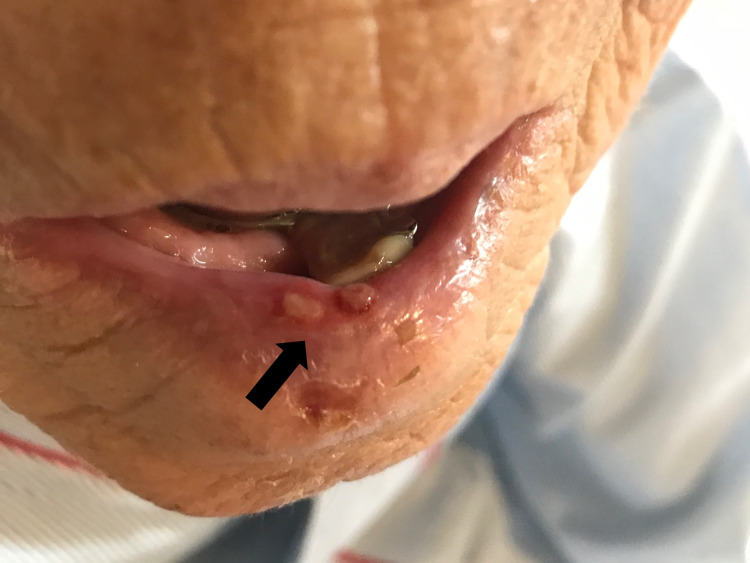
Blisters seen on the patient’s lower lip.

Herpes esophagitis was diagnosed. Because she could not eat anything, she was treated with intravenous acyclovir (750 mg/day). Three days after admission, several blisters appeared on her lower lip (Figure 4).

**Figure 5:**
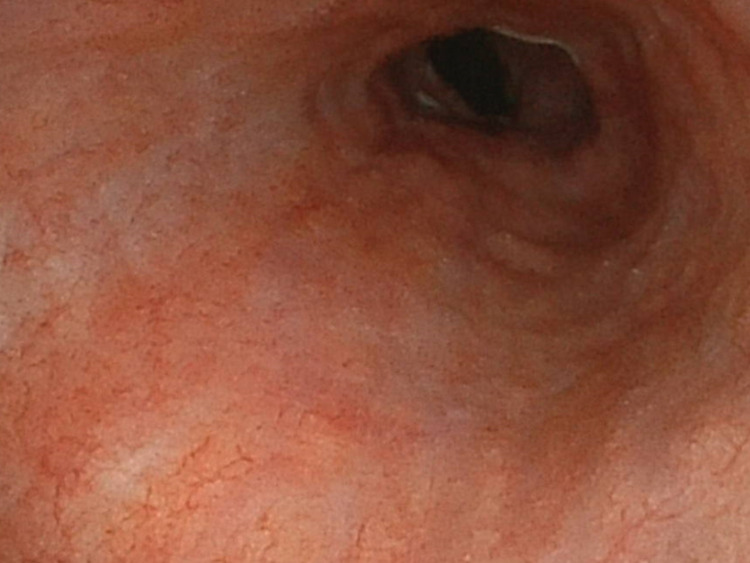
Follow-up of the upper gastrointestinal endoscopy shows the healed mucosa of the esophagus.

After seven days of treatment, her symptoms resolved, and she was discharged to continue her normal life. Follow-up upper gastroscopy showed healed mucosa in the esophagus (Figure 5).

## Discussion

This case shows a vague appearance of herpes esophagitis in an immunocompetent elderly person and suggests that clinicians should consider the possibility of herpes esophagitis among older people with complaints of appetite loss and fatigue.

Herpes esophagitis can be an important differential diagnosis for vague symptoms among older people. It can present with various symptoms, based on previous reports and reviews [1], such as chest pain, nausea, vomiting, and epigastric pain [3]. The symptoms triggered by herpes esophagitis can be caused by the invasion of the mucus and the surrounding nerves. However, some cases can show vague symptoms, such as fatigue, appetite loss, and weight loss. This may be related to atypical symptoms in elderly patients with several diseases [5].

The vague symptoms of herpes esophagitis can be related to weak sensory irritation of the esophageal mucosa. In particular, the lower part of the esophagus does not have pain receptors [6]. Therefore, ulceration of the lower part of the esophagus may not cause severe pain, which can make the symptoms vague. At this time, ulcers and inflammation located in the lower part can match the evidence. In addition, in this case, esophageal inflammation and bleeding might have caused fatigue and appetite loss. Thus, the physiology and anatomy of the esophagus can result in symptoms of herpes esophagitis being vague.

The possibility of herpes esophagitis should be reconsidered in older patients. It is thought to be common in immunocompromised hosts but is rare in immunocompetent hosts [4], including elderly patients [7]. However, because aging is suggested to cause relative immunodeficiency, several immunological preventions such as vaccination against influenza and pneumococcal pneumonia are recommended [8]. Herpes virus infection is related to cell-mediated immunology [1]. Based on the results of previous studies, aging can weaken cell-mediated immunology because of dysfunction or reduced neutrophils [6]. In this case, because the patient was quite old, her cell-mediated immunology could have been affected to the extent of allowing the herpes virus to harm the esophagus. As the older population increases, especially in rural areas, help-seeking behavior is becoming a critical issue [9-11]. Fear of infection during the coronavirus disease 2019 pandemic reduced the rate of older people in rural areas accessing professional care. They have gotten used to receiving comprehensive medical care without needing to go to urban areas [12,13]. Accordingly, rural primary care physicians have to treat a larger variety of symptoms than ever before [14,15]. For clinicians, the diagnosis of herpes esophagitis based on vague symptoms should be performed effectively to improve elderly care.

## Conclusions

This case suggests that herpes esophagitis may lead to vague symptoms in elderly individuals. This disease should be considered as a differential diagnosis not only in immunocompromised hosts but also in elderly people. Clinicians should consider the possibility of herpes esophagitis among older people with complaints of appetite loss, fatigue, and other vague symptoms.
